# Differential regulation of calcium‐activated plant kinases in *Arabidopsis thaliana*

**DOI:** 10.1111/tpj.70413

**Published:** 2025-09-05

**Authors:** Martin Zauser, Anja Liese, Anna Feldman‐Salit, Melanie Krebs, Karin Schumacher, Tina Romeis, Ursula Kummer, Jürgen Pahle

**Affiliations:** ^1^ Biological Information Processing Group, BioQuant Heidelberg University Im Neuenheimer Feld 267 69120 Heidelberg Germany; ^2^ Department of Plant Biochemistry of Interactions Leibniz Institute of Plant Biochemistry (IPB) Halle Germany; ^3^ Department of Modeling of Biological Processes, BioQuant Heidelberg University Im Neuenheimer Feld 267 69120 Heidelberg Germany; ^4^ Department of Cell Biology Centre for Organismal Studies (COS) Heidelberg, Heidelberg University Heidelberg Germany; ^5^ Biochemistry of Plant Interactions Martin‐Luther‐University Halle‐Wittenberg D‐06120 Halle (Saale) Germany

**Keywords:** calcium, signaling, calcium‐dependent protein kinase (CPK), guard cells, flg22

## Abstract

The decoding of calcium signals by plant calcium‐dependent kinases (CPKs) is not fully understood yet. Based on kinetic *in vitro* measurements of the activity of several CPK proteins, their individual activity profile was modeled and coupled to cytosolic calcium concentration changes from *in vivo* measurements of guard cells and epidermal leaf cells. In addition, computationally produced surrogate data were used. It was analyzed how individual activity profiles of CPKs will change in response to the different calcium time courses. In addition, the impact of modeling explicitly individual elementary reaction steps for binding and unbinding in comparison to using a heuristic Hill equation for the binding process was investigated.

## Significance Statement

We model the activity profiles of several plant calcium‐dependent protein kinases (CPKs) based on kinetic *in vitro* measurements and couple these to cytosolic calcium concentration changes from *in vivo* measurements from guard cells and epidermal leaf cells. We then analyze how individual CPKs are activated in response to different calcium time courses, and study the influence of the binding process speed using a heuristic Hill equation or a model based on elementary binding steps.

## INTRODUCTION

Calcium signaling plays an important role in almost all aspects of plant life and orchestrates developmental processes and responses to external abiotic and biotic stress cues. Externally applied stresses may induce changes in the intracellular calcium concentration as rapid as within milliseconds. In response to biotic stress, for example, upon attack by phytopathogenic microbes, the intracellular calcium increase is initiated by receptor‐mediated recognition of the pathogen (Eichstädt et al., 2021; Huang et al., 2019; Keinath et al., 2015; Maintz et al., 2014; Ranf et al., 2011; Thor & Peiter, 2014). In response to abiotic stress, such as high or low temperature, drought, high light, osmotic changes triggered by salt, or by mechanical wounding, a perception mechanism is less clear, and a broader time range for intracellular calcium changes is observed (Bush, 1995; Feijó & Wudick, 2018; Knight et al., 1991; Tian et al., 2020; Xu et al., 2022; Zhu, 2016). Intracellular calcium changes differ in shape, amplitude, and time, and often an oscillatory pattern is observed. These so‐called ‘calcium signatures’ in plants are a composite result of joint activities from calcium channel molecules on one hand, and calcium exporters and pumps on the other hand (Kudla et al., 2018; Tian et al., 2020; Xu et al., 2022). Calcium signatures are stimulus‐specific, allowing coordinated calcium decoding that translates into changes of protein activities, transcriptional reprogramming, and changes in metabolite patterns to ultimately lead to enhanced stress tolerance or pathogen resistance in the plant (Knight et al., 1996; Knight et al., 1997). The shape of the calcium signature depends also on the cell type and developmental state of cells.

The resting cytosolic free calcium concentration in plant cells is around 100 nM (Logan & Knight, 2003) with some variance. In roots, a value of about 48 nM in the early differentiation zone and up to 154 nM in the root tip was reported (Waadt et al., 2017; Wagner et al., 2015). In guard cells (GC), the baseline calcium concentration was determined between 70 nM and a maximum of 250 nM (Allen et al., 1999; Costa et al., 2018; Grabov & Blatt, 1998; Huang et al., 2019; McAinsh et al., 1990; Schroeder & Hagiwara, 1990; Siegel et al., 2009).

Upon stimulation of cells, for example, by pathogen‐associated molecular patterns (PAMP) treatment or abiotic stress stimuli, the cytoplasmic calcium concentration in various plant cell types can temporarily rise to 500 nM, or up to 1000 nM, or even more (Bagur & Hajnóczky, 2017; Clapham, 1995; Grabov & Blatt, 1998; Logan & Knight, 2003; McAinsh et al., 1990; Michard et al., 2008; Schroeder & Hagiwara, 1990; Zayzafoon, 2006). This increase can occur either as a single pulse, multiple pulses, or periodic multiple pulses (oscillations).

Calcium signatures are decrypted by calcium‐binding proteins. In plants, there exist arrays of calcium decoders that often form large gene families. Besides calmodulin, these include the plant‐specific gene families of calcium‐dependent protein kinases (CDPKs, CPKs in *Arabidopsis thaliana*), calcineurin B‐likes (CBLs)/CBL‐interacting protein kinases (CIPKs), and CaM‐like proteins CML (Kudla et al., 2018; Tang et al., 2020; Yip Delormel & Boudsocq, 2019). Among these, the class of CDPKs is unique in containing the calcium sensor and the protein kinase effector domains within the same molecule (Cheng et al., 2002; Yip Delormel & Boudsocq, 2019). Some CDPKs are well characterized both biochemically and in terms of their biological functions, and CDPKs decoding calcium signals related to drought tolerance or pathogen resistance are known (Köster et al., 2022; Kudla et al., 2018). The calcium‐dependent protein activities of these CDPKs provide an ideal entry point for modeling the CDPK‐specific capacity to decode distinct stress‐related calcium signatures.

The model plant *Arabidopsis thaliana* codes for 250 putative EF‐hand calcium‐binding proteins; among these are 34 CPKs (Cheng et al., 2002; Day et al., 2002; Yip Delormel & Boudsocq, 2019). Characteristic of CPKs is a catalytic protein kinase domain that is linked via a short junction domain functioning as a pseudosubstrate region to a regulatory calmodulin‐like domain (CaM‐LD). The CaM‐LD contains four calcium‐binding EF‐hand motifs. The EF‐hands function in pairs, forming an N‐terminal EF lobe and a C‐terminal EF lobe. Additionally, many CDPKs are located at the plasma membrane through an N‐terminal myristoylation and palmitoylation site (Boudsocq et al., 2012; Simeunovic et al., 2016; Yip Delormel & Boudsocq, 2019).

This study investigates the mode of information processing from calcium signatures to enzyme activation of five selected (membrane associated) CDPKs from *Arabidopsis*. CPK5 and CPK6 are activated in response to a PAMP receptor‐mediated increase in the cytosolic calcium concentration in plant mesophyll cells upon exposure to pathogen‐related stimuli (Dubiella et al., 2013; Huang et al., 2020). Both enzymes phosphorylate and activate the membrane‐bound substrate protein respiratory burst oxidase homolog protein (RBOHD) that catalyzes the production of reactive oxygen species (ROS) (Boudsocq et al., 2010; Dubiella et al., 2013; Kadota et al., 2014; Köster et al., 2025). In plant immunity a local recognition of a pathogen leads to the transmission of the information of ‘having been attacked’ to distal unharmed plant parts, enabling systemic resistance against the pathogen (Hake & Romeis, 2019). Mutations in *cpk5cpk6* leading to impaired RBOHD‐dependent ROS‐signaling result in enhanced susceptibility to pathogen attack in systemic tissues (Boudsocq et al., 2010; Dubiella et al., 2013; Guerra et al., 2020). CPK21 and CPK23, but also CPK5, CPK6, and CPK3 are involved in the regulation of membrane‐bound S‐type anion channels such as the guard cell‐specific slow anion channel 1 (SLAC1) mediating stomatal closure (Brandt et al., 2012; Brandt et al., 2015; Geiger et al., 2010; Mori et al., 2006; Scherzer et al., 2012). Measurements of the currents of S‐type anion channels with loss‐of‐function mutants showed that CPK3, CPK6, and CPK23 play an important role in its activation, with CPK6 having a stronger effect than CPK3 (Geiger et al., 2011; Mori et al., 2006). Experiments in *Xenopus* oocytes further reveal that CPK3, CPK5, CPK6, CPK21, and CPK23 can phosphorylate and activate SLAC1 also in that heterologous expression system (Brandt et al., 2012; Brandt et al., 2015; Geiger et al., 2010; Scherzer et al., 2012). These five CPKs have been biochemically characterized as recombinant purified proteins and data from *in vitro* protein kinase assays describing calcium‐dependent phosphorylation activities toward substrate peptides are available (Boudsocq et al., 2012; Geiger et al., 2010; Guerra et al., 2020; Liese et al., 2024).

Due to the complex nature of the coding and decoding modes in calcium signaling, the mechanism behind information processing via dynamic calcium signals has so far in general been studied using computational models based on experimental data ‐ either for animal cells (Smedler & Uhlén, 2014), or on a more conceptual level (Salazar et al., 2008). Only a few reports address directly the processing of calcium signals by calcium/calmodulin binding in transcriptional activation or CCaM kinase activation in plant cells (Lenzoni et al., 2018; Sathyanarayanan & Poovaiah, 2004).

Here, we couple calcium concentration changes *in planta* with calcium sensitivity of CPKs using source data from published kinase activity measurements (Guerra et al., 2020; Liese et al., 2024), as well as newly measured data on CPK3. We take microbe‐derived molecules flg22 and chitin as elicitors that represent conserved PAMP motifs for bacteria (flg22) and fungi (chitin). Previous experiments in *Arabidopsis* leaves have already demonstrated that the external addition of flg22 and chitin leads to immediate transient calcium responses that often are displayed as a series of short pulses (Eichstädt et al., 2021; Keinath et al., 2015; Li et al., 2021; Thor & Peiter, 2014).

To investigate how different plant calcium signatures are processed into distinct CPK isoform activations, we use computational modeling as an integrative approach. Two different models are employed in the analysis: (1) a detailed model based on individual elementary calcium‐binding steps, and (2) a more heuristic model based on Hill kinetics. For both models, we use a combination of experimental and surrogate data. This allows us to assess whether the simpler model based on Hill kinetics is sufficient to describe CPK isoform activation; or whether the detailed modeling of individual calcium‐binding steps is necessary.

So far, such an approach has only been applied in a few studies on calcium signaling and not yet for analyzing calcium signaling in plant cells. In previous non‐plant studies, it was already reported by us that the cooperativity of calcium binding of effector proteins is able to decode various signal forms, such as oscillations, spikes, and bursts (Larsen et al., 2004). In a theoretical study based on that furthermore, it was shown that it is possible to regulate two different proteins differentially with periodic calcium bursts, with one protein being more responsive to high peaks and the other to lower peaks (Schuster et al., 2005). Both studies assume a periodically oscillating calcium signal. In comparison, however, the induced calcium signal in plant cells in response to the exposure of leaves to flg22 or chitin triggers only a few individual calcium pulses and then subsides again (Eichstädt et al., 2021; Keinath et al., 2015; Li et al., 2021; Thor & Peiter, 2014). In one theoretical study, it was possible to selectively activate proteins (Marhl et al., 2006) by changing the width and number of the calcium peaks with a single sequence of calcium pulses. Fast activation and slow deactivation of the respective proteins was particularly important here, allowing the protein to be significantly activated by means of fewer calcium pulses. In another study, the information encoded in the frequency of calcium oscillations (referred to as frequency coding) is analyzed. The authors distinguish between proteins whose activity increases with increasing oscillation frequency (referred to as high‐pass activation) and proteins that exhibit maximal activity at a specific frequency (referred to as band‐pass activation, for example, nuclear factor of activated T cells (NFAT)) (Schoch & Pahle, 2019). In addition to frequency coding, amplitude coding has also been observed, in which an increasing stimulus intensity is encoded by an increase in peak amplitude at a constant frequency (Schweizer et al., 2011). Furthermore, the advantages and disadvantages of frequency and amplitude coding for information processing were discussed (Aguilera et al., 2019).

Here, we use computational models that are based on experimental data to describe the decoding by CDPKs in response to distinct pathogen effector‐induced calcium signals. Exemplified by the five membrane‐bound *Arabidopsis* CPKs described above, heuristic Hill‐like kinetics proves to be sufficient for the modeling of the individual phosphorylation activities. A more detailed mechanistic model comprising elementary calcium‐binding steps is not necessary. The described heuristic Hill model links individual calcium peaks and signatures with CPK phosphorylation activities and allows us to predict whether an individual enzyme is still inactive, active, or again de‐activated in a cellular context.

## RESULTS

### CPKs differ in their calcium‐dependent protein kinase activities in *in vitro* protein kinase (IVK) assays

**Figure 1:**
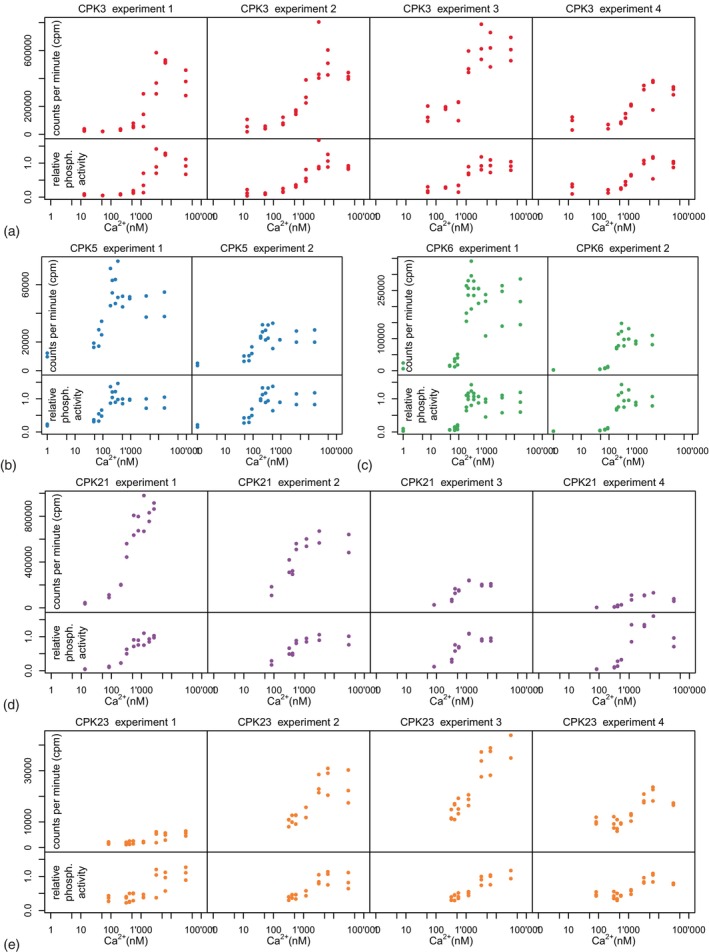
*In vitro* kinase assay data of phosphorylation activity of CPK proteins CPK3 (a) on a SLAC1 peptide, CPK5 (b) and CPK6 (c) on a RBOHD peptide or syntide 2, and CPK21 (d) and CPK23 (e) on a SLAC1 peptide. The upper panels show raw measurement data; the lower panels show relative phosphorylation activity. Two experiments each were carried out for CPK5 and CPK6, and four experiments each for CPK3, CPK21, and CPK23. The dataset of each experiment includes 2–3 technical replicates. For calculating the relative activity, scaling factors were fitted to the experimental data using the parameter estimation task in the COPASI/CoRC software (Förster et al., 2021; Hoops et al., 2006).

To determine the mode of information processing from different plant calcium signatures to distinct CPK activation, five *Arabidopsis* CPKs (CPK3, CPK5, CPK6, CPK21, CPK23) were chosen, for which data from IVK with recombinant proteins were available. These CPKs share either RBOHD or SLAC1 as an *in vivo* substrate, and derived peptides encompassing respective known phosphorylation sites had been used as substrates for IVKs at different calcium concentrations (Figure 1).

Despite rather noisy measurement curves, semi‐quantitative trends become obvious. CPK5 and CPK6 display similar low activity at calcium concentrations up to approximately 50 nM. The activity increased sharply at calcium concentrations between 50 and 300 nM; beyond that, there was no further increase in activity. CPK21 revealed low activity at calcium concentrations up to approximately 300 nM, and the activity increased strongly at calcium concentrations between 300 and 1000 nM, with no further increase above 1000 nM. The activity of CPK3 increased around 1000 nM, with no further increase above 3000 nM. The activity of CPK23 is constant within the physiological range between 100 and 1500 nM of calcium, with higher calcium concentrations only resulting in a slight further increase in activity. However, enzyme activity can be increased up to a calcium concentration of approximately 6000 nM, and only above 6000 nM was no further increase in activity observed.

Some of the IVK assays in this study show an activation “overshoot” for CPK proteins at a calcium concentration of approximately 2000 nM, that is, the activity increases sharply, but then drops again at even higher calcium concentrations. Such behavior has not been observed in related studies so far, nor with similar calcium‐binding proteins in other cells and organisms, and we do not have a readily plausible physiological explanation. Therefore, and in the light of the high variability of the data, we conclude that this effect is related to the experimental protocol and the measurement accuracy. We therefore assumed that there is actually a monotonically increasing activity forming a Hill‐shaped curve. However, this effect also makes the determination of the maximum phosphorylation activity challenging. In particular, the absolute counts‐per‐minute readings differ significantly when the experiment is repeated using the same experimental protocol. We therefore scaled the absolute measured values accordingly and used the relative phosphorylation activity of the respective CPK protein in relation to its maximum activity as a basis for our model simulations as described below. Since the resulting kinetic parameters are similar to parameters previously published and for the purpose of this specific study, we think this is admissible.

In summary, it can be stated that all the proteins examined here display cooperativity and have different operating points. This means that there is a significant difference between, on one hand, the calcium concentration at which the cooperativity leads to a strong activation of the enzyme at only a slight increase in calcium concentration, and on the other hand, the enzyme activity at the basal cytosolic calcium concentration. CPK5 and CPK6 are most sensitive to changes in calcium at around 100 nM (the basal cytosolic concentration), CPK21 at around 500 nM, CPK3 at around 1000 nM, and CPK23 at around 2000 nM (above the normal range of cytosolic calcium). For a plant cell, this means: CPK5 and CPK6 respond very sensitively to concentration changes of calcium, CPK3 and CPK21 only become active when calcium levels are quite high, and CPK23 activity is almost calcium‐independent. CPK23 was reported to reach full IVK phosphorylation activity only at micromolar calcium concentrations, which is apparently irrelevant under *in vivo* conditions.

### *In vivo* calcium signatures are specific to PAMP elicitor and plant cell type

**Figure 2:**
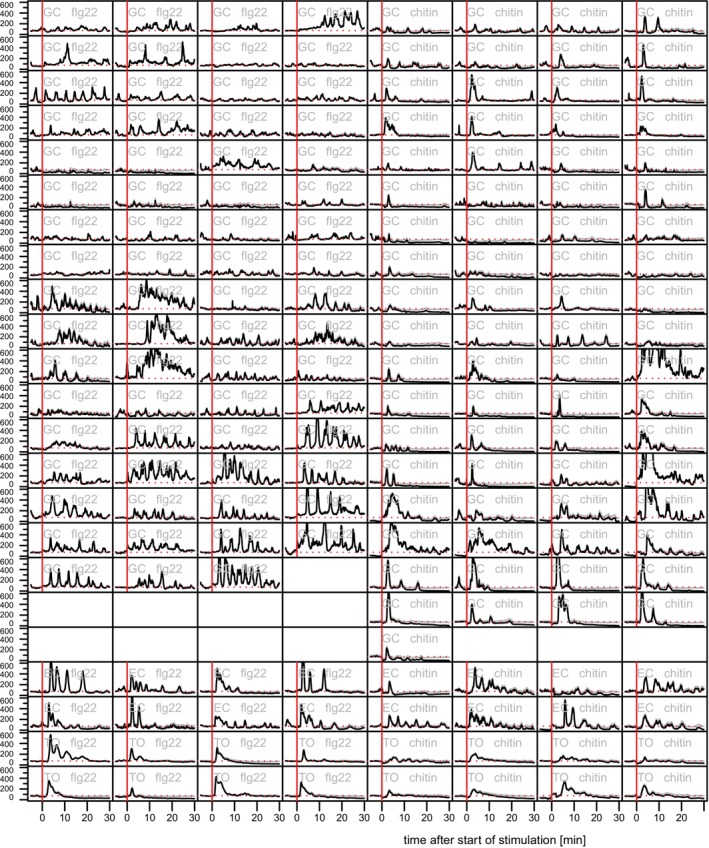
Data sets of the calcium input data stimulated by flg22 or chitin (GC = guard cell, EC = epidermal cell, TO = total region of interest). Raw concentration data (in nM) are shown in black; linearly corrected data (in nM) are shown in gray. Dotted horizontal line marks the baseline at 74 nM. A vertical red line marks the points in time of stimulation.

To allow the modeling of information processing of plant calcium signatures, existing quantitative data derived from *Arabidopsis* leaves expressing the calcium sensor R‐GECO were used as input data (Figure 2). Leaves of seedlings were exposed to the bacterial elicitor flg22 or the fungal elicitor chitin, and respective calcium dynamics were recorded by live cell imaging over time in GC or epidermal cells (EC) (Keinath et al., 2015).

As shown in Figure 2, the form of the calcium signal varies from cell to cell. Nevertheless, some similarities of the signals become obvious. It typically consists of a sequence of 3–8 individual pulses with a pulse width of approximately 2–3 min, and a period of about 5 min. Few cells display a single pulse lasting several minutes or show no noticeable change in concentration at all (Figure 2). Notably, despite significant variability between individual cells, GC exhibit a higher number of calcium peaks and increased oscillation frequency following flg22 stimulation compared with chitin treatment. In contrast, a PAMP‐specific calcium signature is not clearly observed in EC when comparing flg22 and chitin treatments. In total cell populations, which represent a mix of EC and GC, calcium responses are characterized by fewer pulses, often just one or two peaks. These calcium signals likely represent an amalgamation of asynchronous oscillations occurring in individual cells. This observation aligns with previous mathematical models analyzing how calcium responses vary with the number of cells measured (Dodd et al., 2006).

Extracellular calcium was reported to induce oscillations of intracellular calcium with pulses lasting about 25 min, and with the oscillations stopping after 45–60 min (Allen et al., 2000). Therefore, the calcium concentration at the time point of 20 min after elicitation was chosen as a reference for the stimulated state for modeling the information processing. The unambiguous determination of the calcium concentration in the unstimulated state turned out to be challenging. In many cells, the basal calcium concentration is slightly lower after the stimulation than before stimulation. This may be due to the experimental protocol and may not reflect an actual concentration drop in the cell. Because CPK proteins CPK5 and CPK6 are highly calcium sensitively responding in the range of the cellular basal calcium concentration, a reliable determination of the base concentration is required. A linear correction of the measured values was introduced so that in each time series, the mean base concentration after stimulation is identical to the mean base concentration before stimulation (Figure 2).

The experimental calcium time series served as a starting point for computational modeling of the calcium‐induced protein activity in *Arabidopsis* cells. Based on the *in vitro* kinase assay data, first a model for the phosphorylation activity of the CPK proteins was created. Then, the experimental calcium time series was coupled as an input to the CPK model.

### Modeling the CPK activity to increased levels of intracellular calcium

**Figure 3:**
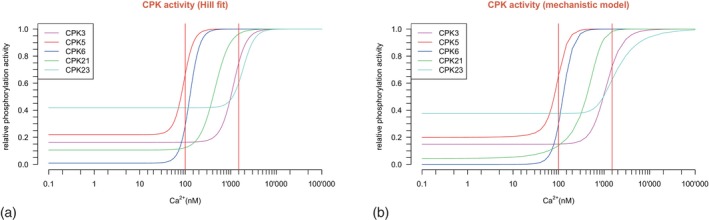
Hill model (a) and mechanistic model (b) of CPK protein activity in response to intracellular calcium. Vertical red lines mark the active range of intracellular calcium between 100 and 1500 nM.

**Figure 4:**
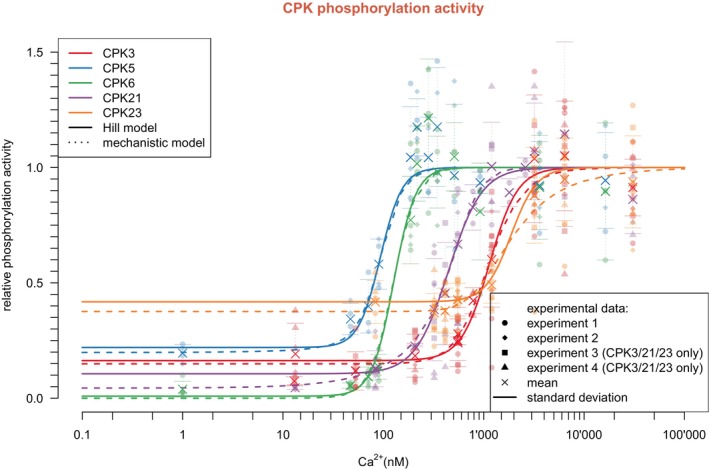
Experimental data of CPK phosphorylation activity in response to intracellular calcium measured by IVK assays were fitted by the computational models. CPK response curves of the Hill models are depicted by solid lines; curves of the mechanistic model are depicted by dotted lines. All measured data points of the experiments are marked by small, semi‐transparent symbols. The mean of all measurement values for a given calcium concentration is marked by a cross; horizontal lines mark the standard deviation.

The simulation of kinase activity was implemented by two types of mathematical models: a heuristic Hill type model and a detailed model incorporating the elementary chemical binding reactions (Figures 3 and 4).

#### Hill model

All analyzed CPKs reveal a clear sensitivity to calcium changes within the physiological range of intracellular calcium concentration (Figure 3a). Assuming cooperativity in calcium binding, we performed a parameter estimation for the relative protein activity f based on the Hill equation using the COPASI/CoRC software (Förster et al., 2021; Hoops et al., 2006):

$$f=\frac{v_{\max}*S^{h}}{K_{m}^{h}+S^{h}}+\text{basal},$$

with $v_{\max}$ the maximal relative protein activity, S the calcium concentration, h the Hill coefficient, $K_{m}$ the half maximal constant and basal the basal activity without the presence of calcium.

Based on sequence data, CPK3, CPK5, CPK6, and CPK21 reveal four binding sites for calcium. CPK23 also has four binding sites; however, one binding site deviates from the conserved EF‐hand structure and may be dysfunctional. Assuming a maximum of four binding sites are available, for the Hill coefficient an upper limit was set to four. Fitting the kinetics to the experimental data resulted in a Hill coefficient between 2.6 for CPK21 and 4 for CPK5 and CPK6, indicating a positive cooperativity of the calcium‐binding sites (Table 1). CPK5 has a very high affinity to calcium, and CPK3 has a low affinity to calcium, confirming previous results (Boudsocq et al., 2012). CPK23 displays low affinity to calcium with a calcium‐independent activity of 42%. This is in line with an earlier study reporting high affinity to calcium for CPK21 and low affinity and a 60% calcium‐independent activity for CPK23 (Geiger et al., 2010).

**Table 1:** Parameter sets for modeling the relative phosphorylation activity of different CPK proteins in response to increasing calcium based on fitting a Hill equation to *in vitro* assay data. The Hill coefficient was limited to a maximum of 4. No further restrictions were applied

Protein	*K*_*m*_ [nmol l^−1^]	*v*_max_ [relative activity]	*h* (Hill coefficient)	basal (calcium‐independent base activity) [relative activity]
CPK3	1139	0.8377	3.1053	0.16323
CPK5	93.236	0.78006	4	0.21994
CPK6	126.83	0.99107	4	0.008932
CPK21	446.2	0.89418	2.6051	0.10582
CPK23	1877.5	0.58232	3.4053	0.41768

#### Mechanistic model

In addition to using the Hill function as a proxy for the calcium‐induced activity of the respective enzymes, a mechanistic model based on the elementary binding processes was generated (Figure 3b). It was assumed that the affinity change of the next binding site due to the cooperativity after the binding of one calcium ion to the first binding site is so fast that it can be considered to occur instantaneously. Therefore, the affinity of the binding sites for calcium has been modeled using a Monod Wyman Changeux (MWC) model approach. In that model, the CPK protein is represented by five different species: the apoprotein without calcium, and protein variants with one, two, three, or four bound calcium ions. The transition from one intermediate to the next happens sequentially, that is, first one calcium ion binds, then another, etc. It was further assumed that a calcium ion always binds to the free binding site with the highest affinity for calcium, so that the individual binding sites do not become further distinguished. The binding of the calcium ion has been modeled with a binding and an unbinding reaction. The overall system therefore consists of eight reaction equations:

$$\begin{array}{c} \frac{d\left(\left[{\mathrm{CPK}}_{\mathrm{ca}0}\right]\right)}{\mathrm{dt}}=-k_{\mathrm{on}1}\cdot \left[{\mathrm{CPK}}_{\mathrm{ca}0}\right]\cdot \left[\mathrm{Ca}\right]+k_{\mathrm{off}1}\cdot \left[{\mathrm{CPK}}_{\mathrm{ca}1}\right]\\ \frac{d\left(\left[{\mathrm{CPK}}_{\mathrm{ca}1}\right]\right)}{\mathrm{dt}}=+k_{\mathrm{on}1}\cdot \left[{\mathrm{CPK}}_{\mathrm{ca}0}\right]\cdot \left[\mathrm{Ca}\right]-k_{\mathrm{on}2}\cdot \left[{\mathrm{CPK}}_{\mathrm{ca}1}\right]\cdot \left[\mathrm{Ca}\right]\\ \;-k_{\mathrm{off}1}\cdot \left[{\mathrm{CPK}}_{\mathrm{ca}1}\right]+k_{\mathrm{off}2}\cdot \left[{\mathrm{CPK}}_{\mathrm{ca}2}\right]\\ \frac{d\left(\left[{\mathrm{CPK}}_{\mathrm{ca}2}\right]\right)}{\mathrm{dt}}=+k_{\mathrm{on}2}\cdot \left[{\mathrm{CPK}}_{\mathrm{ca}1}\right]\cdot \left[\mathrm{Ca}\right]-k_{\mathrm{on}3}\cdot \left[{\mathrm{CPK}}_{\mathrm{ca}2}\right]\cdot \left[\mathrm{Ca}\right]\\ \;-k_{\mathrm{off}2}\cdot \left[{\mathrm{CPK}}_{\mathrm{ca}2}\right]+k_{\mathrm{off}3}\cdot \left[{\mathrm{CPK}}_{\mathrm{ca}3}\right]\\ \frac{d\left(\left[{\mathrm{CPK}}_{\mathrm{ca}3}\right]\right)}{\mathrm{dt}}=+k_{\mathrm{on}3}\cdot \left[{\mathrm{CPK}}_{\mathrm{ca}2}\right]\cdot \left[\mathrm{Ca}\right]-k_{\mathrm{on}4}\cdot \left[{\mathrm{CPK}}_{\mathrm{ca}3}\right]\cdot \left[\mathrm{Ca}\right]\\ \;-k_{\mathrm{off}3}\cdot \left[{\mathrm{CPK}}_{\mathrm{ca}3}\right]+k_{\mathrm{off}4}\cdot \left[{\mathrm{CPK}}_{\mathrm{ca}4}\right]\\ \frac{d\left(\left[{\mathrm{CPK}}_{\mathrm{ca}4}\right]\right)}{\mathrm{dt}}=+k_{\mathrm{on}4}\cdot \left[{\mathrm{CPK}}_{\mathrm{ca}3}\right]\cdot \left[\mathrm{Ca}\right]-k_{\mathrm{off}4}\cdot \left[{\mathrm{CPK}}_{\mathrm{ca}4}\right] \end{array}.$$

The individual protein variants with 0 to 4 bound calcium ions are represented by CPK_ca0_ to CPK_ca4_. $k_{\mathrm{on}1}$ to $k_{\mathrm{on}4}$ are the binding constants of calcium, $k_{\mathrm{off}1}$ to $k_{\mathrm{off}4}$ are the unbinding constants. It has been assumed that the intermediates have different phosphorylation activity. The protein without calcium has the lowest activity, the protein with four calcium ions has the highest activity, which has been equated with the value 1. It also has been assumed that the activity increases as the number of bound calcium ions increases. The activity of the single intermediates has been described using a weighting parameter $w_{i}$, where $w_{4}$ is set to 1 by definition. The relative phosphorylation activity of a CPK protein can then be calculated as the ratio of the weighted sum of the concentration of the intermediates to the total concentration of protein:

$$\text{activity}=\frac{\sum_{i=0}^{4}w_{i}\cdot \left[{\mathrm{CPK}}_{\mathrm{cai}}\right]}{\sum_{i=0}^{4}\left[{\mathrm{CPK}}_{\mathrm{cai}}\right]}$$

A separate set of parameters has been generated for each of the five CPKs (Table 2). For this purpose, a parameter estimation using the experimental steady‐state data has been performed. Since in the steady state only the ratio between the rate of binding and unbinding can be determined, but not the individual rates, the binding rates $k_{\mathrm{on}1}$ to $k_{\mathrm{on}4}$ at 1 have been fixed, and only the unbinding rates have been estimated (dissociation constants).

**Table 2:** Parameter sets for modeling the relative phosphorylation activity of different CPK proteins in response to increasing calcium based on differential equations. $\;k_{\mathrm{on}}$ rates were set to 1 $s^{-1}\cdot nM^{-1}$ for all binding reactions. The $k_{\mathrm{off}}$ rates were limited within a range of 10 $s^{-1}$ to 1000 $s^{-1}$. The weights $w_{0}$ to $w_{4}$ are within a range of 0 to 1 with $0\leq w_{0}\leq w_{1}\leq w_{2}\leq w_{3}\leq 1$. $w_{4}$ was set to 1 assuming that the fully occupied protein has the maximal activity

Protein	*k*_off1_ [s^−1^]	*k*_off2_ [s^−1^]	*k*_off3_ [s^−1^]	*k*_off4_ [s^−1^]	$w_{0}$	$w_{1}$	$w_{2}$	$w_{3}$	$w_{4}$
CPK3	1000	1000	1000	771.59	0.14892	0.14927	0.15159	1	1
CPK5	1000	894.0	10	10	0.1983	1	1	1	1
CPK6	1000	1000	24.1	10	1.7e‐39	5.3e‐29	1	1	1
CPK21	1000	1000	970.9	109.0	0.0439	0.9871	1	1	1
CPK23	1000	1000	1000	935.4	0.3758	0.3758	0.3758	0.37643	1

The kinetics of kinase activity modeled by the Hill model or the mechanistic model are shown in Figures 3 and 4. The Hill model corresponds to a mechanistic model with instantaneous response. In this model, the CPK activity immediately follows the input. In the mechanistic model, the CPK activity depends on the speed of the reactions. Remarkably, both modeling approaches yielded similar results, with a close correlation between both models for CPK5 and CPK6, enzymes of high calcium affinities, and slight deviations at the high concentration range for CPKs of lower affinity, in particular also for CPK23. These data indicate that the Hill model may suffice to describe CPK decoding, and a more detailed mechanistic model may not be necessary.

Next, the experimental data originating from IVK phosphorylation activities in response to intracellular calcium for all five CPKs were used to fit the parameters of both computational models, the Hill model and the mechanistic model, and compiled in Figure 4.

### CPK response to artificial generated calcium signals

**Figure 5:**
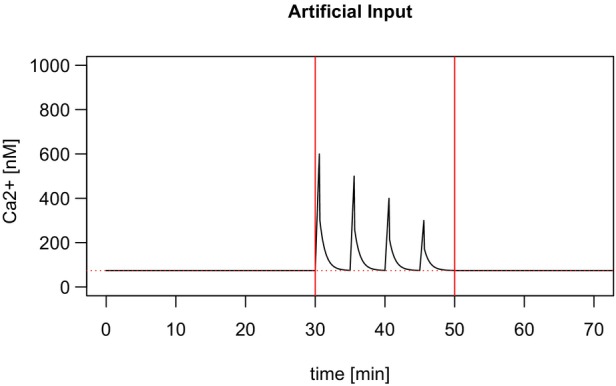
Artificial calcium data consisting of four single triangular pulses with exponential decay. The dotted horizontal line marks the baseline at 74 nM. Horizontal lines mark the points in time for calculating the mean calcium concentration after stimulation (time 30–50 min).

To further validate the models and to investigate the influence of the calcium signal on the CPK protein activity in a strictly defined manner, artificial calcium signals were generated. As exemplarily depicted in Figure 5, a calcium signature of four short triangular pulse trains of different intensities and of decreasing amplitude was generated. A calcium concentration of 600 nM has been chosen as the (maximum) amplitude.

**Figure 6:**
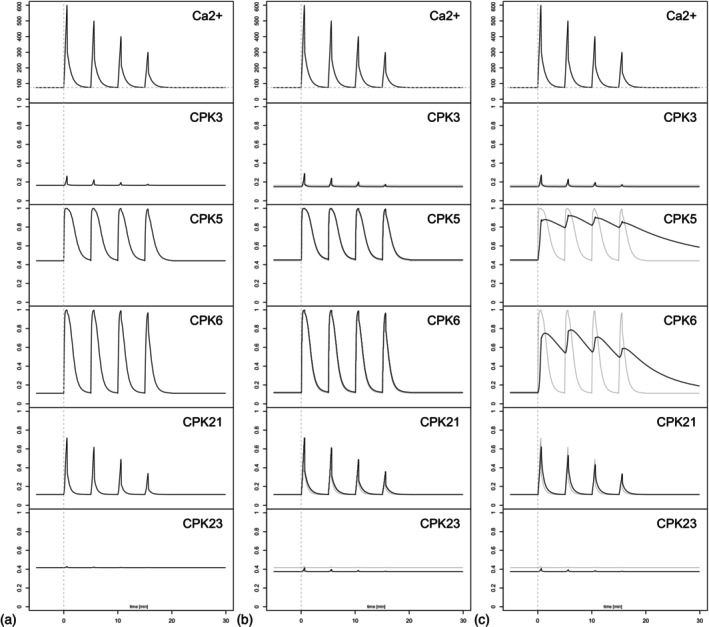
Artificial calcium time series were used as an input to our models. Using these input data for simulations based on the Hill model reveals that the protein activity here is a direct response to the calcium input (a). Simulations of the mechanistic model were performed with different reaction speeds: default speed (b) and low speed, slower by a factor of 1000 (c), with the Hill model shown in gray. The base level for the artificial input time series is 74 nM.

These artificial calcium time series were fed as a time‐dependent input into the CPK models for the different CPK proteins and simulated with the Hill model and the mechanistic model. Notably, the Hill model corresponds to a mechanistic model with instantaneous response (Figure 6a). For the mechanistic model, all five CPKs were simulated with default speed of the program (Figure 6b) and low speed, slower by a factor of 1000 (Figure 6c).

Two major observations can be seen in the CPK response to these artificial calcium signals (Figure 6). (1) Fast binding and unbinding of the calcium ions to the enzyme lead to the same behavior as the coupling of a Hill model to the calcium signal (panels a and b). Slow binding, in contrast, results in different behavior, in particular observed for CPK5 and CPK6 (panel c). For these enzymes, slow binding does not allow the enzyme activity to return to the basal state between individual amplitudes of the oscillatory signal. Therefore, an overall increased average activity of the enzyme is the consequence. (2) Different CPK isoforms show distinct decoding properties in all cases. Depending on their basal activity and steepness of the Hill curve, some kinases (like CPK3 and CPK23) are hardly activated at all in response to these artificial calcium signals, whereas other CPKs such as CPK5, CPK6, and CPK21 respond with remarkable sensitivity to the calcium time series.

### Calcium‐mediated differential regulation of CPKs in *Arabidopsis* plants

**Figure 7:**
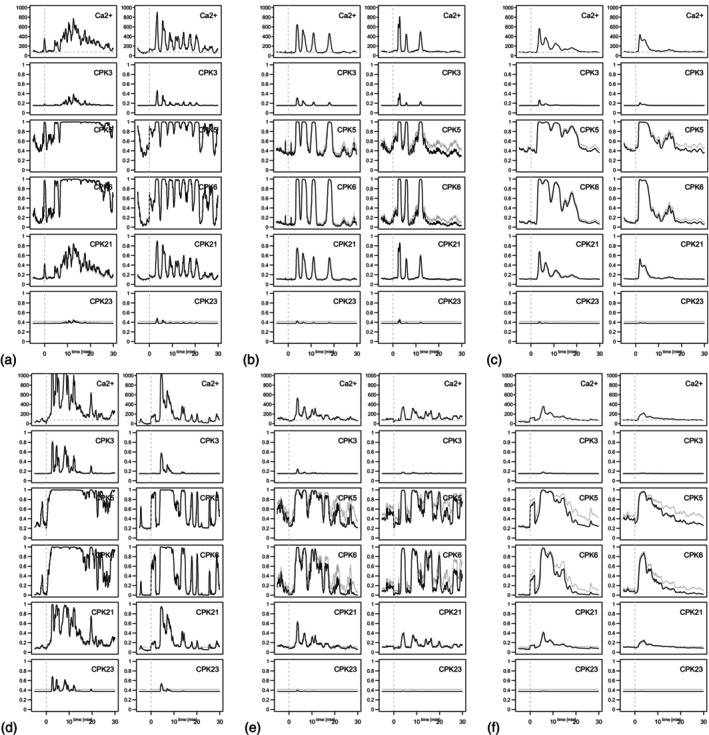
Results of example simulations of dynamic CPK activity data in response to calcium signaling. Calcium data from a stimulation experiment with flg22 (a, b, c) and chitin (d, e, f) were used as input, in which guard cells (a, d), epidermal cells (b, e), and the plant cells in total (c, f) were analyzed. Several time series of calcium concentration values were recorded for each of these six groups. As an example, the two data sets with the highest calcium increase per group were selected after calculating the averaged raise in calcium after stimulation (one dataset is depicted in the left column, the other in the right column of each panel). The graphs in the first row of each panel show the time course of the calcium concentration in nM. The graphs in rows 2–6 of each panel show the simulation results of the relative phosphorylation activity of the CPK proteins CPK5, CPK6 toward RBOHD, and CPK3, CPK21, and CPK23 toward SLAC. The beginning of the stimulation with the elicitor is represented by a vertical dashed line (time point 0 min). For a complete overview of the calcium input datasets, see Figure 2.

Next to the artificial calcium signature, the generated CPK models were applied in stimulations using a realistic calcium time series as input that originates from live cell imaging of *Arabidopsis* leaves stably expressing the calcium sensor R‐GECO1 (Keinath et al., 2015, Figure 2). Simulations of dynamic activities for all five CPK isoforms in response to flg22 or chitin elicitation were performed using respective calcium time series of either GC, EC, or total plant cells (which represent a mix of EC and GC). The data were processed as described in the Methods. Again, the CPK dynamic activities were simulated with Hill (shown in gray) and the mechanistic model with default speed (shown in black) (Figure 7).

Similar to the results when coupling artificial calcium time series, also here the activity of the CPKs follows the calcium concentration. CPK3 and CPK23 are only activated by the strongest calcium signals, and this only to a very limited extent. This is in line with a recent study using a newly developed genetically encoded fluorescent biosensor for CPK conformational activation, which displays that CPK21‐FRET, but not CPK23‐FRET, can report calcium oscillations in pollen tubes (Liese et al., 2024). However, also for CPK21, it becomes obvious that weaker calcium signals lead to no significant activation; whereas, CPK5 and CPK6 are very sensitive to all calcium signals. Even more, for high‐frequency oscillations, for example, flg22‐treated GC (panel a), when the calcium concentration decreases to resting levels, the enzyme activity does not return to the basal state between individual peaks of the oscillatory signal.

While general trends are expected due to the knowledge of the kinetics of calcium activation, it is convenient to be able to follow the activation in detail for different calcium signals.

**Figure 8:**
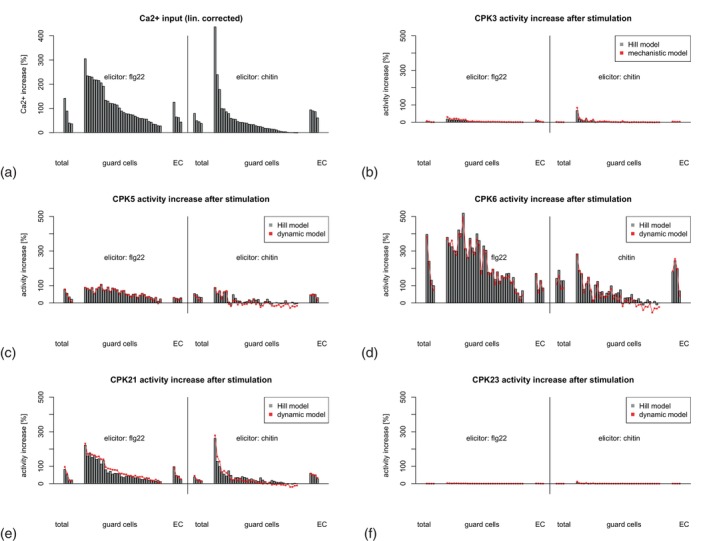
CPK activity increase in response to flg22 and chitin in guard cells (GC) and epidermal cells (EC). The increase of the relative phosphorylation activity was calculated as the ratio between base activity (mean concentration of calcium between time point 20 min before start of stimulation and time point of stimulation) and activity after stimulation (mean concentration of calcium between time point start of stimulation and 20 min after start of stimulation). Panels: Ca^2+^ concentration increase (a), increase in activity of CPK3 (b), CPK5 (c), CPK6 (d), CPK21 (e), and CPK23 (f) after stimulation. Left hand side elicitor flg22 and right hand side elicitor chitin.

The kinase activity obtained can be considered as the sum of the activity increase during the decoding period (here 20 min after treatment) relative to the activity before treatment (Figure 8). This sum of activity may result in a sum of target phosphorylation in the decoding period. The observed slight changes in CPK3 and CPK23 kinase activity simulated for individual calcium peaks are reflected by a minor summarized activity increase over the decoding period (panel b and f). Despite comparable overall calcium level changes, the summarized activity increase during the decoding period in GC is for CPK5 and CPK6 more pronounced after flg22 than chitin treatment (panel c and d). CPK21 activity increase is comparable between flg22 and chitin decoding (panel e). In summary, Figure 8 shows that the simulation leads to a different increase in activity in the individual CPKs.

## DISCUSSION

The CPK proteins investigated here contain four EF motifs as calcium‐binding sites and are therefore able to respond to an increase in the calcium concentration in the cell with a change in conformation and a successive change in their activity. Thereby, the activity follows cooperatively with respect to the calcium concentration. The steady‐state measurements with the substrate peptides, for example, SLAC1, reveal the differences between the individual CPK proteins in calcium dependency. The proteins differed both in their relative phosphorylation activity in the absence of bound calcium and in the concentration of calcium at which phosphorylation activity is clearly enhanced by calcium binding. In addition, the strength of the cooperativity varied between the enzymes. CPK5, CPK6, and CPK21 show a high sensitivity to calcium. In contrast, CPK3 and CPK23 are rather insensitive and can only be activated beyond their basal activity by high concentrations of calcium ions. In the case of CPK23, this is presumably due to the protein structure of one of the four EF‐hands of CPK23 deviating from the consensus sequence (Liese & Romeis, 2013). It could be shown for several *Arabidopsis thaliana* isoforms that degenerated EF‐hand motifs are connected to low or no calcium sensitivity (Boudsocq et al., 2012). For CPK23 kinase activity, a switch in calcium sensitivity in dependence on an auto‐phosphorylation site within the junction domain of CPK23 has been reported (Liese et al., 2024). Mutation of this site preventing auto‐phosphorylation shifts CPK23 calcium sensitivity to a more physiologically relevant calcium range, indicating that dephosphorylation of CPK23 may prime CPK23 for calcium‐dependent kinase activity increment. It may seem surprising that in this case this phosphorylation event does not reside within the calcium‐binding domain but is located in the junction domain (CPK23). This implies that the conformational change of CPKs initiated by the binding of calcium may be regulated additionally by phosphorylation‐dependent changes through inter‐domain interactions. Nevertheless, to couple *in vivo* calcium changes to CPK target phosphorylation, a model based on cooperative calcium‐binding events initiating directly kinase activity proves a good approximation to the *in vivo* situation.

Regarding the *in vivo* calcium signature shown in Figure 7, it is striking that depending on the signal frequency, CPK5 and CPK6 activity does not return to basal levels between individual peaks, even when calcium concentrations reach resting basal levels. This phenomenon is especially pronounced during high‐frequency oscillations, which are characteristic of guard cells (GC), and less pronounced in epidermal cells (EC) (see Figures 2 and 7).

Notably, despite a significant variability between individual GC, the number of peaks and the oscillation frequency tend to be higher following flg22 stimulation compared with chitin treatment (Figure 2). The observed lack of CPK5 and CPK6 deactivation during high‐frequency oscillations suggests that their activity increases with oscillation frequency (i.e., frequency coding), even when the amplitude of calcium changes remains similar (i.e., amplitude coding). Interestingly, both CPK5 and CPK6 exhibit a more substantial activity increase upon flg22 treatment than with chitin in GC, despite comparable overall calcium level changes (Figure 8). Biologically, the sustained activity of CPK5 and CPK6 during oscillations may result in enhanced phosphorylation of downstream targets such as SLAC1 and RBOHD. In this context, a recent study is of particular interest, which utilized a blue light activated calcium channel in GC (Huang et al., 2024). This study demonstrated that the number of light‐induced calcium peaks determines both the extent and speed of stomatal closure. However, when the interval between pulses is too long (e.g., 60 sec), the correlation between the number of calcium peaks and the rate of stomatal closure is partly disrupted. Additionally, the authors were able to link individual calcium peaks to time‐correlated anion currents, likely associated with SLAC1 activation. Thus, our here presented modeling approach, which identifies and describes a frequency‐dependent lack of deactivation in certain kinases upstream of the anion channel SLAC1, provides an explanation of how GC “count” the number of calcium peaks. In this study, only the relative activity was measured; that is, the maximum activity was used to normalize the activity measurements in dependence on calcium. Nevertheless, when comparing the proteins with one another, large differences in phosphorylation activity became evident. The plant cell can use this differential regulation of the CPK proteins as a tool to adapt the reaction controlled by the phosphorylated substrate, for example, the closing of the stomata by the GC in response to the stress reaction underlying the calcium signal, such as drought or attack by pathogens. So, the CPK proteins can be viewed as adjusting screws with which the plant's reaction to a stress situation can be regulated.

Our modeling of the CPK protein activity using the Hill model and comparing it to a detailed model based on elementary binding steps shows that the Hill model is able to well describe the activity profile of the individual CPKs as long as the binding and unbinding of the calcium ions is fast or in the defined “medium” range.

There are only a few measurements of exact binding and unbinding kinetics available, for example, for calmodulin (Faas et al., 2011), but in all of these measured cases, the binding and unbinding are very fast (Table 3). Thus, when analyzing the range of realistic binding and unbinding constants of calcium to CBPs, these lie well within the fast binding described above, indicating that a heuristic model describing the decoding is absolutely sufficient. However, it is noteworthy that for proteins that would exhibit slower calcium binding, one would have to resort to the more detailed model in order to fully grasp the behavior during the processing of fast calcium dynamics in the plant cell.

**Table 3:** $k_{\mathrm{on}}$ and $k_{\mathrm{off}}$ rates of different calcium‐binding proteins

Protein	*K*_*d*_ [nM]	*k*_on_ [nM^−1^ s^−1^]	*k*_off_ [s^−1^]	Comment/reference
EGTA	71	0.0105	0.7455	Nägerl et al. (2000)
calbindin‐D28K (3:1)	175	0.013	2.275	Nägerl et al. (2000)
calbindin‐D28K (3:1)	513	0.077	39.501	Nägerl et al. (2000)
calbindin‐D28K (2:2)	237	0.011	2.607	Nägerl et al. (2000)
calbindin‐D28K (2:2)	411	0.087	35.757	Nägerl et al. (2000)
BAPTA	160	0.1–1	16–160	Naraghi (1997)
Oregon‐Green‐BAPTA 1 (OGB‐1)	170	1	170	Faas and Mody (2012)
CPK model default	10–1000	1	10–1000	
CPK model low speed	10–1000	0.001	0.01–1	

## CONCLUSIONS

CPKs function as decoders of calcium signals in plant signaling and represent signaling hubs in the control of plant development, abiotic stress tolerance, and pathogen resistance. Likewise, CPKs as decoders are also drivers of calcium signal propagation from local to distal parts of the plant. Here, models for information processing are provided that allow the prediction of individual activity profiles of individual CPK isoforms in response to cytosolic calcium concentration changes. These models have been developed based on kinetic *in vitro* kinase measurements of kinase activities toward two selected peptide substrates, SLAC1 and RBOHD, and, obviously, they are quantitatively only valid in this context. However, expecting that the *in vivo* kinetics will not differ enormously, these are actually capable of validly describing the behavior of CPKs in a much wider context of responses to calcium signals in plant cells – at least qualitatively. In particular, when employing the heuristic Hill function, the coupling of these computational kinetic models to experimental data may work well as long as binding rates of calcium are in the range of what has been described as fast. The employment of a more detailed model describing binding and unbinding events yet will have to be used for much slower binding rates. Both of these models could be easily and quickly adjusted to take additional factors regulating the activity of the CPKs into account.

It is well‐known that decoding of calcium signals in nature involves concerted action of multiple calcium‐binding proteins of different nature and classes. Thereby, decoders may become additionally modified by post‐translational modifications, such as phosphorylation. These modifications may lead to different kinetic parameters of the individual enzymes, and the mode of decoding (inactive, active, de‐activated) in response to defined calcium signals may be changed. Furthermore, in the context of biological function, calcium decoders such as CPKs show differential localization (plasma membrane vs. cytosol), are expressed in different plant cells and tissue (root, shoot; epidermal cell vs. guard cell), and are accordingly phosphorylating different *in vivo* substrates displaying different substrate‐specific activation kinetics. Thus, extensions of the models will be needed to provide a more holistic view on information processing *in planta*. In any case, the current models allow the integration of future quantitative measurements and knowledge in a straightforward way.

## METHODS

### IVK analysis of differential regulation of CPK phosphorylation activity

The raw data of the kinase assays were derived from already published kinase activity measurements (Guerra et al., 2020; Liese et al., 2024), except for CPK3 which was newly measured. Detailed description of the published *in vitro* kinase measurements can be found in the respective publication. Similarly, for CPK3 activity measurements, the coding region of CPK3 was cloned into the pET30a expression vector (Novagen) with an additional C‐terminal StrepII tag. The expression of the CPK3 protein and the affinity purification using the StrepII‐tag were described elsewhere (Guerra et al., 2020). For StrepII purification, the following buffers were used: lysis buffer (100 mM Tris–HCl (pH 8.0), 150 mM KCl, 100 μg/mL Avidin, 1 mM DTT, 1:100 protease inhibitor cocktail for histidine‐tagged proteins); wash buffer (100 mM Tris–HCl (pH 8.0), 150 mM KCl); and elution buffer (100 mM Tris–HCl (pH 8.0), 250 mM KCl, 2.5 mM Biotin). Purity and concentration of HIS‐ and GST‐purified CPK21 and CPK23 (Liese et al., 2024) and StrepII‐purified CPK3, CPK5, and CPK6 were analyzed via 10% SDS‐PAGE and Coomassie staining, as well as by the Bradford protein concentration assay. The purified CPK3 protein was dialyzed as described by Liese et al. (2024), with the modification that the dialysis buffer contained 45 mM MOPS instead of 30 mM.

*In vitro* kinase measurements were conducted for CPK3, CPK21, and CPK23 using a substrate peptide from the *in vivo* substrate SLAC1 (amino acids 41–60, encompassing S59: RGPNRGKQRPFRGFSRQVSL), and for CPK5 and CPK6 using an RBOHD substrate peptide (amino acids 141–150, encompassing S148: RELRRVFSRR).

For CPK3 kinase reactions (30 μL), the kinase was incubated in a buffer containing 37.5 mM MOPS (pH 7.4), 125 mM KCl, 10 mM MgCl_2_, 10 μM ATP, 3 μCi [γ‐^32^P]‐ATP, 10 μM substrate peptide, 6.67 mM EGTA, and varying concentrations of CaCl_2_. For CPK21 and CPK23, the final MOPS concentration in the kinase assay reaction mix was 25 mM instead of 37.5 mM (Liese et al., 2024). Kinase reaction mixes for CPK5 and CPK6 contained 125 mM NaCl instead of 125 mM KCl and 38.3 mM Tris (pH 8.0) instead of 37.5 mM MOPS (pH 7.4) (Guerra et al., 2020).

The reaction mixtures were incubated for 20 min at 22°C and stopped by the addition of 3 μL of 10% phosphoric acid. Radiolabeled phosphorylation of the substrate peptides was determined after binding to P81 filter paper and scintillation counting; as described by Franz et al. (2011).

Calcium‐dependent kinase activity is indicated as percentage of maximal activity mean value of all calcium concentration grouped means of technical replicates equal or larger than the mean value of technical replicates at the largest calcium concentration (“dynamic maximum”). Kinase activities can be described by a four‐parameter equation based on $K_{m}$, $v_{\max}$, Hill coefficient and basal activity (Hill model).

### Stimulation of *Arabidopsis thaliana* plants by flg22 or chitin

Calcium signal changes in response to flg22 and chitin in leaves of 14‐ to 16‐day‐old seedlings were calculated by analyzing fluorescence images of R‐GECO1 in the top imaging mode with upward‐facing leaf abaxial epidermis as described in Keinath et al. (2015). Image acquisition was started 30 min before stimulation. For stimulation, leaves were treated with 100 nM flg22 or 100 μg/mL chitin. Concentration values were calculated from normalized R‐GECO1 fluorescence intensities (Δ F/F) according to the formula described in Wang et al. (2015, figure S2B).

#### Calcium data measurement

Time series data were collected from eight samples of stimulation with flg22 and 8 samples of stimulation with chitin. For each sample, the fluorescence intensity was analyzed in several regions of GC, in one epidermal region, and in the total image. In total, there are 67 data sets for flg22/GC, 73 data sets for chitin/GC, and 8 data sets each for flg22/EC, chitin/EC, flg22/total, and chitin/total.

Data acquisition started 30 min before stimulation and was continued for 40 min after stimulation, resulting in an unstimulated period from time 0 to time 30 min and a stimulated period from time 30 min to time 70 min. Frame rate was 5 sec.

#### Calcium data preprocessing

Fluorescence raw data were converted into concentration values by linear scaling of the data assuming a basal unstimulated concentration of intracellular calcium of 74 nM. The corresponding fluorescence value was calculated as the median of all measurement values of a period of 20 min before stimulation (time 10 –30 min). Linear scaling was assumed due to reports on the corresponding dynamics range of R‐GECO1 in the nM range, reaching saturation only in the μM range (Akerboom et al., 2013; Wang et al., 2015).

To compensate for bleaching of the calcium sensor, the data sets were increased linearly if the concentration at the end of the experiment (median of all values from time 60 – 70 min) was lower than at the beginning of the experiment (median of all values from time 10 min to time 30 min). In addition, an artificially created input data set with four small triangular peaks was used. Figure 2 shows example data sets.

It was often difficult to reliably determine the baseline concentration in our measurement data. In particular, a different baseline concentration after stimulation was often observed. We assumed that this shift in the baseline concentration is an effect of the experimental setup and therefore corrected the baseline concentration mathematically.

#### Calcium data selection

Because not all cells responded to the stimulation with an increase in the intracellular calcium concentration and calcium signals were already visible in some cells before the stimulation, a preselection of the calcium data sets was made for the simulation of the calcium‐induced CPK activation. For this, data sets were scored by calculating the increase in intracellular calcium as the ratio of the mean concentration 20 min after stimulation to the mean concentration in the time period 20 min before stimulation. Data sets were sorted by the concentration increase. The 50% with the lowest scores were discarded.

### Computational setup

The algorithms for analyzing the CPK protein activity were developed using the open‐source programming language R (version 4.2.1) on macOS Catalina (version 10.15.7) on a MacBook Pro with an Intel 2.5 GHz Quad‐Core i7 processor and a Windows‐PC with an Intel i5 CPU with 1.6 GHz (R Core Team, 2023). For modeling the activity of the CPK proteins, the open‐source software COPASI (COmplex PAthway SImulator; version 4.29 build 228) was used in combination with the corresponding R package CoRC (version 0.11.0) which provides a convenient developer's API for the biochemical modeling tools of COPASI (Förster et al., 2021; Hoops et al., 2006). COPASI/CoRC was also used to fit the experimental data by the Hill model. Parameter estimations were run on a compute cluster with Linux (CentOS 6.4 × 86 64‐bit Kernel 2.6.32) using Sun Fire AMD Opteron and IBM Quad Intel Xeon CPUs.

## Author Contributions

UK and JP planned and designed the research. AL and MK performed experiments and analyzed data; TR and KS were involved in discussions and experimental design. MZ and AFS created models and analyzed simulation data. UK, JP, and MZ wrote the manuscript. All authors read and edited the manuscript.

## Data Availability

The data that support the findings of this study are available on request from the corresponding author. The data are not publicly available due to privacy or ethical restrictions.
